# Stress and its association with academic performance among dental undergraduate students in Fujian, China: a cross-sectional online questionnaire survey

**DOI:** 10.1186/s12909-020-02095-4

**Published:** 2020-06-03

**Authors:** Xiu-Jiao Lin, Chang-Yuan Zhang, Song Yang, Ming-Lun Hsu, Hui Cheng, Jiang Chen, Hao Yu

**Affiliations:** 1 Department of Prosthodontics, School and Hospital of Stomatology, Fujian Medical University, Fuzhou, China; 2 Department of Dentistry, School of Dentistry, National Yang-Ming University, Taipei, Chinese Taipei; 3 Department of Implantology, School and Hospital of Stomatology, Fujian Medical University, Fuzhou, China; 4 Department of Applied Prosthodontics, Graduate School of Biomedical Sciences, Nagasaki University, Nagasaki, Japan

**Keywords:** Dental students, Stress, Academic performance, China

## Abstract

**Background:**

The aim of this study was to investigate the amount and sources of stress in dental undergraduate students in Fujian, China, and the factors associated with stress.

**Methods:**

This cross-sectional study was conducted during the second semester of the 2017–2018 academic year at the School of Stomatology, Fujian Medical University, China. A total of 396 students were surveyed with the Dental Environment Stress Questionnaire (DES) and the Perceived Stress Scale (PSS) using an online survey system. The participants’ demographic information, including sex, age, year of study, and grade point average (GPA) was also collected. One-way analysis of variance (ANOVA) was performed to compare the stress scores. Pearson correlation and multiple linear regression analyses were conducted to explore the associated factors of stress and academic performance. All statistical analyses were performed at a significance level of 5%.

**Results:**

A total of 347 undergraduate students participated in the present study, for a response rate of 87.6%. There were no significant differences in the DES and PSS total scores among students of different grades and sexes. Significant differences were found in the DES “workload” and “self-efficacy beliefs” scores among students from different study years (all *P* < 0.05). The Multiple linear regression showed that DES and PSS scores were negatively correlated with GPA, while sex was positively correlated with GPA (all *P* < 0.05). Female students had significantly higher GPAs than male students.

**Conclusions:**

Dental undergraduates in Fujian, China experienced moderate levels of stress. While the amount of stress did not differ by year of study, the sources of stress did differ. Stress scores and sex were negatively correlated with academic performance.

## Background

The term “stress” describes external demands, either physical or psychological, on an individual’s physical and mental health [1–3]. Stress is not just a stimulus or a response; rather, it is a process of perceiving and coping with environmental events [4]. Perceived and potential stress affect students relatively often, making them vulnerable to psychological problems and impacts on their physical well-being [1]. Previous findings have suggested that dental school is a highly stressful environment and have shown that dental students present higher levels of stress than medical students and the general population [5–7]. Dental students need to demonstrate both excellent academic performance and precise clinical skills during their studies, which results in a high level of stress [8]. The reported causes of stress (stressors) in dental students vary but are mainly related to living accommodations, the teaching curriculum, and academic and clinical work [8–12].

Numerous researchers have evaluated stress in dental students using various instruments [1, 2, 9, 11, 13–15]. The most frequently employed instruments are the Dental Environment Stress Questionnaire (DES) and the Perceived Stress Scale (PSS) [1]. The DES, developed by Garbee [16] in 1981, was designed to describe stressors related to predoctoral dental training, whereas the PSS was designed to assess short-term general perceived stress. It has been reported that people with different social-cultural backgrounds, learning experiences, and sexes may perceive the same stressors differently [4, 10, 13, 17, 18]. Coping strategies and examination-related self-efficacy have also been suggested to play a role in stress [19]. Although studies using the DES and/or PSS have been performed predominantly in North American, European, and some Asian countries [1, 6, 9, 13, 15–17, 20], very limited information is available for other countries. As the largest developing country in the world, China has a significant shortage of dental professionals [21]. Although the total number of dentists and assistant dentists in China increased from 129,504 in 2013 to 167,227 in 2016 [22], the current dentist/population ratio (0.12:1000) is still far below the WHO’s ideal level (1:7500) [23]. In response to this lack of dentists, the enrollment of undergraduate dental students has increased dramatically in China in recent decades. Unfortunately, to the authors’ knowledge, no report on stress in Chinese dental students has been published.

Academic performance is critical for academic success, which makes students more competitive in the job market and strongly predicts their social and occupational success in the future [24]. Moreover, academic performance is also important due to the expectations of one’s elders and a desire to outperform one’s peers. While stress provides stimulation that up to a point can be considered academic motivation, prolonged stress can lower performance and lead to health problems [2]. In the literature, controversial findings have been reported regarding the correlation between stress and students’ academic performance. Some studies showed that students reporting higher stress had lower grade point averages (GPAs) [2, 19, 25], whereas another study found no correlation between stress and academic performance among Australian dental students [26]. The role that stress plays in academic performance may depend on the level of stress [27]. Nevertheless, a greater understanding of the amount and sources of stress as well as the associations of stress with academic performance among Chinese dental students is critical for dental educators.

Therefore, the objectives of the present study were 1) to investigate the amount and sources of stress experienced by Chinese dental undergraduate students using the DES and PSS; and 2) to explore the factors associated with stress.

## Methods

### Ethical approval

Ethical approval was obtained from the institutional review board of the School of Stomatology, Fujian Medical University (approval no. 2017-JXGG-01). A trained researcher was personally responsible for recruiting participants and providing them with information about the purpose of the study.

### Study design and setting

A cross-sectional study was conducted in Fujian Province, which is located in the southeast region of China and has a surface area of 121,400 km^2^. This study was carried out among all the preclinical dental undergraduate students attending Fujian Medical University, China. Fujian Medical University is currently the only university providing dental undergraduate and postgraduate education and training in Fujian Province. The Bachelor of Dental Surgery (BDS) degree in China is a 5-year program in which the first to fourth years are preclinical and the fifth year is clinical.

### Participants

Participants were recruited from among registered preclinical (first- to fourth- year) undergraduate students at the School of Stomatology, Fujian Medical University. The sample size was estimated based on a formula by Cochran: when the population is 600 (total number of dental undergraduate students in Fujian Medical University), the approximate sample size should be 235 with a margin of error of 0.05 and a critical value of 1.96 [28]. Therefore, the sample size was set at 396, assuming a response rate of 60%. Students were invited to participate in this study during the second semester of the 2017–2018 academic year and were informed that participation was voluntary and anonymous. The questionnaire was distributed to 396 undergraduate students through an online survey system (http://www.wjx.cn). A cover letter explaining the study design, the consent form and the importance of the study were provided to reduce the nonresponse bias.

### Study variables

The participants’ GPAs, which ranged from 0 to 4, were chosen as the outcome data. The age, sex, year of study, and stress scores of participants were selected as the associated factors in the present study.

### Data collection

Data on the age, sex, year of study, marital status, GPA, and stress of participants were collected by a self-report survey. Stress in dental undergraduate students was measured using the DES and PSS. Data were collected from November 2018 to February 2019.

### Study instrument

The DES used was adapted from the original 38-item DES, which assesses sources of stress related to undergraduate coursework and training in dental students [1, 9] (Supplementary Table l). Six items related to clinical training were omitted because they were not relevant to preclinical students. The 32 items were clustered into 5 domains of potential stressors: “social stressors” (items 1–10), “faculty and administration” (items 11–16), “workload” (items 17–22), “self-efficacy beliefs” (items 23–27), and “performance pressure” (items 28–32). Respondents were asked to rate each item based on their experience using a 4-point Likert scale: 1 = not stressful, 2 = slightly stressful, 3 = moderately stressful, and 4 = very stressful. For nonapplicable items, a fifth response (“not pertinent”) was included. Currently, there is no validated DES in the Chinese language. The questionnaire was translated into Chinese by a native English-speaking bilingual translator and then revised by a native Chinese-speaking bilingual translator. The questionnaire was then back-translated and verified against the original English questionnaire by another native Chinese-speaking bilingual person [17, 29].

The PSS is a 10-item questionnaire on students’ feelings and thoughts during the past month that assesses general perceived stress [30]. Respondents were asked to rate the frequency with which they experienced certain feelings and thoughts using a 5-point Likert scale: 0 = never, 1 = almost never, 2 = sometimes, 3 = fairly often, and 4 = often. The PSS scores were computed, with the responses to the 4 positively stated items (items 4, 5, 7 and 8) reverse scored (e.g., 0 = 4, 1 = 3, 2 = 2, 3 = 1, and 4 = 0). A PSS total score was then calculated for the 10 items. A Chinese version of the PSS was previously validated [31]; therefore, no translation was performed.

The internal consistency (Cronbach’s alpha) for the PSS and DES were 0.81 and 0.86, respectively, indicating the good internal consistency of the questionnaires. The content validity of the questionnaires was confirmed by expert evaluation.

### Data analysis

Statistical analyses were performed using PASW Statistics for Windows Version 18. Descriptive analysis was carried out for the demographic data. One-way analysis of variance (ANOVA) and post hoc Tukey’s HSD tests were performed to compare the DES and PSS scores and to evaluate the sources and level of stress by study year. An independent *t*-test was performed to compare the DES and PSS scores and to compare the sources and level of stress between male and female students. Pearson correlations of the DES and PSS scores with age, sex, gender, year of study, and GPA were calculated. The associated factors of GPA were tested by means of 2 series of multiple linear regression analyses. GPA was regressed on age, sex, year of study, and DES scores (model 1), while GPA was regressed on age, sex, year of study, and PSS scores (model 2). Moreover, multiple linear regression was used to explore whether age, sex, year of study, and/or stressors were associated with the stress scores (models 3 and 4). All statistical analyses were performed at a significance level of 5%.

## Results

### General characteristics of the participants

A total of 347 undergraduate students participated in the present study, for a response rate of 87.6%. Among the 347 students, there were 132 male students (38%) and 215 female students (62%), and the participants had an average age of 20.43 years. All participants were single.

### GPA

The mean GPA of all the participants was 2.86. A significant difference in GPA was found between male and female students (2.72 vs 2.96; t = 2.95, *P* < 0.01; 95% CI of the difference 0.08–0.40).

### PSS

The mean PSS score of all participants was 18.06 (range: 4–33). No significant differences were found in the PSS scores by year of study or sex (Table 1). The top 3 causes of stress that the participants reported were as follows: “often think about things [they] have to accomplish” (mean PSS score: 2.31 (0.85)), “often felt nervous and stressed” (mean PSS score: 2.02 (0.78)), and “often felt that things were going [their] way” (mean PSS score: 1.99 (0.81)).

**Table 1:** PSS scores for all participants

Variable	N	Mean	SD	*P*-value
Year of study				
First year	86	18.04	4.63	0.285
Second year	89	18.77	3.95	
Third year	87	17.41	4.71	
Fourth year	85	17.93	4.90	
Sex				
Male	132	18.58	5.06	0.627
Female	215	17.74	4.20	
Overall	347	18.06	4.55	

### DES

The mean DES score of all participants was 52.21 (range: 0–128). No significant differences were found in the DES total scores by year of study or sex (Table 2). The mean score for each domain of the DES was calculated. Significant differences were found in the DES “workload” and “self-efficacy beliefs” scores among the students in different study years (*P* = 0.015 and 0.020, respectively). The third- and fourth-year students exhibited significantly higher DES “workload” scores than the first- and second-year students. Second-, third-, and fourth-year students had stronger self-efficacy beliefs than first-year students (Table 3). Table 4 shows the mean scores for each DES item by year of study. Significant differences were found among the students in different study years with respect to financial responsibilities (*P* = 0.011), rules and regulations of the dental school (*P* = 0.005), lack of input in the decision-making process in dental school (*P* = 0.009), lack of time for relaxation (*P* = 0.036), amount of assigned course work (*P* < 0.001), lack of time to do assigned school work (*P* = 0.002), uncertainty about dental career (*P* = 0.001), lack of confidence to become a successful dentist (*P* = 0.044), competition for grades (*P* = 0.001), and fear of failing a course of the year (*P* = 0.001).

**Table 2:** DES scores for all participants

Variable	N	Mean	SD	*P*-value
Year of study				
First year	86	49.20	19.47	0.306
Second year	89	52.55	19.75	
Third year	87	52.64	17.66	
Fourth year	85	54.57	16.96	
Sex				
Male	132	51.53	18.76	0.594
Female	215	52.64	18.54	
Overall	347	52.21	18.53

**Table 3:** Mean score (SD) for each DES category by year of study

DES category	Year of study				*P*-value
	First year	Second year	Third year	Fourth year	
Social stressors	13.67 (6.01)	14.54 (6.11)	12.45 (6.07)	13.84 (6.16)	0.149
Faculty and administration	8.30 (4.45)	9.20 (4.17)	9.38 (4.01)	9.89 (3.74)	0.083
Workload	9.51 (4.63)	10.60 (5.19)	10.95 (3.94)	11.69 (4.01)	0.015 (4 > 1,2,3)
Self-efficacy beliefs	8.63 (4.07)	9.56 (3.78)	10.33 (3.95)	9.04 (3.15)	0.020 (2,3,4 > 1)
Performance pressure	8.51 (4.41)	9.36 (4.52)	9.84 (3.51)	10.11 (3.89)	0.060
Total score	49.20 (19.47)	52.55 (19.76)	52.64 (17.66)	54.57 (16.96)	0.306

> indicates statistical significance (*P* < 0.05)

**Table 4:** Mean score (SD) for each DES item by year of study

Stressor	First year	Second year	Third year	Fourth year	*P*-value
1. Moving away from home	1.38 (1.05)	1.36 (0.86)	1.16 (0.89)	1.26 (1.07)	0.411
2. Lack of home atmosphere	1.30 (0.93)	1.39 (1.04)	1.20 (0.96)	1.24 (0.95)	0.555
3. Environment in which to study	1.49 (1.06)	1.67 (1.03)	1.63 (1.15)	1.79 (0.93)	0.309
4. Making friends	1.67 (0.96)	1.81 (0.95)	1.68 (1.03)	1.60 (0.90)	0.545
5. Intimate relationships	1.23 (1.34)	1.25 (1.25)	1.01 (1.00)	1.16 (1.11)	0.549
6. Conflict with spouse/partner over career development	0.63 (0.97)	0.80 (0.98)	0.59 (0.81)	0.67 (0.98)	0.516
7. Having multiple roles	1.38 (1.04)	1.43 (1.14)	1.18 (1.13)	1.19 (1.17)	0.337
8. Personal physical health	1.45 (0.93)	1.76 (1.00)	1.57 (1.18)	1.71 (1.06)	0.205
9. Financial responsibilities	1.81 (1.06)	1.92 (0.99)	1.89 (0.98)	1.47 (0.95)	0.011 (1,2,3 > 4)
10. Discrimination due to gender or social class	1.31 (1.02)	1.15 (0.78)	1.04 (0.81)	1.33 (1.03)	0.099
11. Expectation vs reality of dental school	1.66 (1.10)	1.84 (0.99)	1.71 (1.11)	1.69 (0.95)	0.678
12. Approachability of staff	1.01 (0.99)	0.93 (0.92)	0.98 (0.82)	1.07 (0.96)	0.794
13. Criticism about academic or preclinical work	1.41 (1.16)	1.53 (1.05)	1.83 (1.11)	1.62 (1.11)	0.080
14. Rules and regulations of the dental school	1.42 (0.99)	1.62 (1.05)	1.75 (0.98)	1.95 (0.94)	0.005 (2,3,4 > 1)
15. Amount of cheating in school	1.12 (0.89)	1.21 (0.82)	1.24 (0.88)	1.40 (0.88)	0.195
16. Lack of input in decision making process in dental school	1.69 (1.01)	2.07 (1.04)	1.87 (0.91)	2.15 (0.95)	0.009 (4 > 1,2,3)
17. Lack of time for relaxation	1.91 (0.98)	2.21 (0.98)	2.15 (0.99)	2.33 (0.93)	0.036 (4 > 1,2,3)
18. Having reduced holidays compared with other students	1.55 (0.95)	1.69 (1.01)	1.55 (0.89)	1.53 (0.96)	0.680
19. Amount of assigned course work	1.42 (1.04)	1.62 (1.17)	1.93 (1.03)	2.19 (0.82)	< 0.001 (4,3 > 2,1)
20. Lack of time to do assigned school work	1.55 (1.10)	1.62 (1.26)	1.98 (0.83)	2.05 (0.97)	0.002 (4,3 > 2,1)
21. Learning precision manual skills required for clinical and laboratory work	1.26 (1.04)	1.45 (0.98)	1.26 (0.81)	1.33 (0.92)	0.498
22. Late ending time/completing graduation requirements	1.84 (1.11)	2.01 (1.13)	2.08 (0.99)	2.27 (1.09)	0.074
23. Language barrier	1.37 (1.19)	1.25 (1.10)	1.14 (1.01)	1.22 (1.00)	0.556
24. Fear of not being able to catch up if falling behind	2.12 (1.13)	2.27 (1.05)	2.36 (1.01)	2.34 (0.98)	0.418
25. Lack of confidence to be a successful dental student	2.14 (1.22)	2.29 (1.26)	2.36 (1.20)	2.01 (1.04)	0.222
26. Uncertainty about dental career	0.87 (1.02)	0.98 (0.99)	0.66 (0.82)	1.28 (1.11)	0.001 (4 > 1,2,3)
27. Lack of confidence to become a successful dentist	1.17 (0.90)	1.37 (1.00)	1.61 (1.08)	1.34 (1.04)	0.044 (2,3,4 > 1)
28. Difficulty of course work	1.59 (0.96)	1.82 (1.14)	1.82 (0.93)	2.00 (0.99)	0.075
29. Examinations	2.33 (1.20)	2.65 (1.04)	2.74 (1.02)	2.68 (0.95)	0.058
30. Competition for grades	1.95 (1.14)	2.37 (1.09)	2.48 (0.97)	2.54 (1.05)	0.001 (2,3,4 > 1)
31. Fear of failing a course of the year	1.83 (1.11)	1.97 (1.06)	2.38 (1.01)	2.34 (1.03)	0.001 (3,4 > 1,2)
32. Fear of not having possibility to pursue a postgraduate dental education program	1.77 (1.06)	1.96 (1.12)	2.02 (0.99)	2.00 (1.07)	0.378

> indicates statistical significance (*P* < 0.05)

There were no significant differences in the DES scores for the 5 domains between the male and female students (Table 5). Moreover, no significant differences were found in the mean scores for each DES item by sex (all *P* > 0.05).

**Table 5:** Mean score (SD) of each DES category for male and female students

DES category	Sex		*P*-value
	Female	Male	
Social stressors	13.64 (6.05)	13.62 (6.16)	0.970
Faculty and administration	8.99 (4.07)	9.32 (4.16)	0.478
Workload	10.41 (4.59)	10.86 (4.49)	0.373
Self-efficacy beliefs	9.10 (4.11)	9.58 (3.59)	0.255
Performance pressure	9.29 (4.26)	9.55 (4.06)	0.562
Total score	51.53 (18.76)	52.64 (18.54)	0.594

### Correlations between stress and associated factors

Stress scores were significantly and inversely correlated with GPA (r = − 0.119, *P* = 0.029 for the DES; r = − 0.116, *P* = 0.037 for the PSS). There was no significant correlation between stress scores and age, stress scores and sex, or stress scores and year of study (all *P* > 0.05).

The multiple linear regression indicated that stress was significantly and negatively correlated with academic performance. Both the DES and PSS scores were significantly associated with GPA, with a higher stress score predicting a lower GPA. Sex was significantly and positively correlated with GPA, with female students having better GPAs (Table 6). In addition, stressors were significantly correlated with the DES scores (*P* all < 0.001), whereas no significant correlation was found between the demographic data (sex, age, and year of study) and stress scores (Table 7).

**Table 6:** GPA regressed on sex, age, year of study, and stress scores

	Β (Unstandardized B)	S.E.	β (Standardized B)	95% CI for B	*P-*value	R^2^
Model 1 GPA regressed on sex, age, year of study, and DES score						0.047
Sex	0.244	0.082	0.162	0.082 ~ 0.405	0.003	
Age	0.039	0.051	0.077	−0.062 ~ 0.139	0.449	
Year of study	− 0.087	0.066	− 0.131	− 0.217 ~ 0.044	0.191	
DES score	− 0.005	0.002	−0.117	− 0.009 ~ 0.000	0.030	
Model 2 GPA regressed on sex, age, year of study, and PSS score						0.053
Sex	0.244	0.072	0.188	0.103 ~ 0.386	0.001	
Age	0.013	0.045	0.030	−0.075 ~ 0.101	0.769	
Year of study	−0.035	0.058	−0.062	−0.150 ~ 0.079	0.546	
PSS score	−0.016	0.008	−0.114	−0.031 ~ − 0.001	0.040	

Model 1: *P* = 0.003; Model 2: *P* = 0.002. Sex was coded as 1 (male) or 2 (female). Model 1 and Model 2 were run separately

**Table 7:** DES/PSS regressed on sex, age, year of study, and stressors

	Β (Unstandardized B)	S.E.	β (Standardized B)	95% CI for B	*P-*value	R^2^
Model 3 DES score regressed on sex, age, year of study, and stressors						0.954
Sex	−0.399	0.460	−0.010	−1.304 ~ 0.506	0.387	
Age	−0.260	0.289	−0.020	−0.829 ~ 0.309	0.370	
Year of study	0.161	0.383	0.010	−0.592 ~ 0.914	0.674	
Social stressors	7.619	0.423	0.277	6.786 ~ 8.451	0.000	
Faculty and administration	5.984	0.402	0.249	5.194 ~ 6.774	0.000	
Workload	6.002	0.411	0.253	5.193 ~ 6.810	0.000	
Self-efficacy beliefs	5.006	0.378	0.220	4.261 ~ 5.750	0.000	
Performance pressure	5.057	0.327	0.242	4.413 ~ 5.701	0.000	
Model 4 PSS score regressed on sex, age, and year of study						0.016
Sex	−0.678	0.523	−0.073	−1.708 ~ 0.351	0.196	
Age	0.467	0.325	0.151	−0.174 ~ 1.107	0.152	
Year of study	−0.765	0.422	−0.189	−1.596 ~ 0.066	0.071	

Model 3: *P* < 0.001; Model 4: *P* = 0.152. Sex was coded as 1 (male) or 2 (female). Model 3 and Model 4 were run separately

## Discussion

The present findings indicate that the stress experienced by this sample of dental undergraduate students in Fujian, China, was moderate. Moreover, stress in dental undergraduate students predicted lower GPA. Performance pressures, self-efficacy beliefs, and workload were the top stress-provoking factors in dental undergraduate students in Fujian, China.

Attending dental school is considered stressful for students, and this issue has gained increasing attention in the field of education [5–7]. A high prevalence of stress has been observed among dental students in both Western countries [18, 19, 32] and Asian countries [15, 17]. Unfortunately, no such study has been performed in China to date. This study can be considered the first report regarding the stress of Chinese dental undergraduate students based on well-established instruments, namely, the DES and PSS, which makes the results comparable to those of previous reports. Fifth-year dental students were excluded from the study population due to the discrepancy in stress levels between preclinical and clinical students [33].

Although no consensus has been reached regarding cutoffs for the PSS [34], the current literature suggests that a PSS score higher than 20 points indicates high stress [35, 36]. The present results, which showed a mean PSS score of 18.06, indicated that dental students in Fujian, China, experienced moderate stress, which was somewhat lower than expected [19]. The top cause of perceived stress was that the participants “often think about things [they] have to accomplish”, which is in accordance with a previous study [17]. Jacob et al. [37] reported that relative to senior-year students, first-year students presented excessive perceived stress. Although no significant difference was found in the PSS scores among students from different study years, the mean PSS scores were slightly higher for the first- and second-year students.

In the absence of a threshold for DES scores, an average score of 2 or higher could suggest the presence of elevated stress levels [1, 38]. Various DES scores have been reported in the literature. A study conducted in the northeastern United States revealed that dental students perceived higher stress than medical students (score of 81.3 vs. 75.1 on the 34-item DES) [5]. However, a previous longitudinal study that recruited 296 students from 4 U.S. dental schools reported DES and PSS scores of 55.1 and 14.6, respectively [2], which is consistent with the present results. Based on the present findings, it can be suggested that moderate levels of stress are experienced by dental students in Fujian, China, especially when their stress levels are compared with those presented in previous reports from other countries [5, 39]. The novel educational philosophies applied by the examined dental school, such as problem-based learning (PBL), competency-based teaching, and virtual reality programs, may be associated with the lower level of perceived stress [21, 40]. A recent multicountry study recruited 3568 dental students from 14 different dental schools located in Brazil, Croatia, Egypt, India, and Nepal [11]. That authors noted that the strongest stressor was “workload”, with a score of 2.05 (0.56), and the weakest stressor was “social stressors”, with a score of 1.13 (0.65). Similar results were observed in the present study; the domain that was associated with the highest stress was “performance pressure”, followed by “self-efficacy beliefs”, “workload”, and, finally, “social stressors”. More specifically, examinations, a fear of not having the opportunity to enroll in postgraduate dental education program, competition for grades, and uncertainty about their dental careers were the most stress-provoking factors for Chinese dental students. This finding is not surprising, because preclinical students have been reported to have high levels of stress related to “self-efficacy beliefs”, “workload”, and “clinical training” [8, 41], while intrinsic academic motivation has been observed to be negatively associated with depression and stress [32]. Finally, social stressors were found to be the least stressful, which is in agreement with previous studies [9–11].

It is critical for dental schools to identify potential sources of stress to address them effectively. The mean stress level of the Chinese preclinical dental students was higher for those with more years of attendance, but no significant difference was found. However, the sources of stress showed some differences. More specifically, a significantly higher stress level related to “workload” was found for the third- and fourth-year students than for the first- and second-year students, while a significantly higher stress level related to “self-efficacy beliefs” was found for the second- to fourth-year students than for the first-year students. The design of the curriculum may offer a partial explanation for the abovementioned findings [14, 17, 42]. In the first 2 years, undergraduate students at the School of Stomatology, Fujian Medical University, focus on basic sciences (e.g., biomedical sciences, humanities, and social sciences), as in other schools of stomatology in China. Starting in the third year of study, basic and clinical dental sciences are incorporated into the curriculum. In addition t didactic lectures, laboratory training and clinical clerkships are incorporated as students progress through their studies. Toward the fourth year of study, students become more anxious about the transition from the preclinical to the clinical phase and their future prospects [15, 43]. Therefore, the senior (third- and fourth-year) students reported the greatest amount of stress associated with the amount of assigned course work, lack of time to do assigned school work, competition for grades, and fear of failing a course of the year. Moreover, due to the increased workload, senior students lack time for relaxation that could help relieve stress. These findings will enable dental schools and educators to promote stress-coping strategies and modify teaching curricula to reduce students’ stress. Interestingly, although significant differences were found by year of study, based on a comparison of the present findings with data from other countries, dental students in Fujian, China, appeared less concerned about their professional futures than students from other countries [8]. This finding may be a reflection of the relatively low dentist/population ratio in China.

In the literature, female students have been reported to experience higher [2, 41, 42, 44, 45], lower [46], or similar [47] levels of stress as male students. In agreement with Humphris et al. [47], no overall differences in stress were observed between the sexes in the present study.

Regression analyses showed that stress might be an associated factor of academic performance, which is in line with previous studies [2, 19, 48–50]. A negative correlation between stress and GPA was previously shown (*r* = − 0.17, *P* = 0.006 for the DES; *r* = − 0.11, *P* = 0.042 for the PSS) [2], which was consistent with the present findings (r = − 0.119, *P* = 0.029 for the DES; r = − 0.116, *P* = 0.037 for the PSS). It is possible that stress that leads to depression and anxiety with or without physical symptoms could immobilize students, making them unable to deal with the demands of a difficult academic curriculum [51]. Stress levels could be expected to further negatively affect students’ well-being, career options, and lifestyle choices [6, 52]. Nevertheless, the present findings should be interpreted with caution since the small coefficient detected for the association between stress and GPA may be a result of random noise, and the relationship between stress and academic performance might be bidirectional [50, 53]. As mentioned above, stress is a double-edged sword that can either motivate students to enhance their performance or reduce their chances of success [17]. Importantly, the DES item “Lack of time for relaxation” was highly ranked by all the participants. Thus, stress management efforts such as time management, encouragement from advisors and regular exercise are recommended [19].

In addition to stress, sex was found to be associated with academic performance in this study. Female students had significantly higher GPAs than male students, which was consistent with a previous study [19]. However, a study conducted in Israel reported that sociodemographic variables such as gender were not related to the academic performance of therapy students [37]. The different study populations may account for this discrepancy.

The present study has several limitations. First, the present findings were based on a single-center, cross-sectional study comparing students from different years in the program. It is possible that schools using different methods of teaching may have students with different stress levels. Findings based on students at one school may not be useful for extrapolating the situations of students at other schools. In addition, concerning the sample size, although the sample size was appropriate based on our calculation and was within the same range as the sample sizes used in previous studies, it could have limited the power of the regression analyses involving a number of the associated factors. Therefore, replication of this study with larger samples would be advisable. Second, coping strategies and examination-related self-efficacy have been reported to play a role in stress and academic performance in dental students [19]. However, the current study recruited students from different years of study. Different exams may involve different coping strategies and examination-related self-efficacy [19]. Therefore, coping strategies and examination-related self-efficacy were not investigated in this survey. Future research should clarify this issue with samples of students in the same university year.

## Conclusions

In the present study, the top stress-provoking factors in dental undergraduate students were performance pressure, self-efficacy beliefs, and workload. The findings showed that stress levels negatively predicted the academic performance of dental undergraduate students. Measures are needed to reduce stress for improved academic performance of the students. Further research should focus on developing and evaluating the effects of stress-reducing strategies in dental undergraduate students.

## Supplementary information

**Additional file 1: Supplementary Table 1.** 32-item DES questionnaire used in this study.

## Data Availability

Further data may be requested by contacting the corresponding author. We declare that any data regarding the study will willingly provided.

## Abbreviations

DES: Dental Environment Stress Questionnaire
PSS: Perceived Stress Scale
ANOVA: Analysis of variance
