# Supplementary material for: Stress and its association with academic performance among dental undergraduate students in Fujian, China: a cross-sectional online questionnaire survey
Source: BMC Med Educ. 2020 Jun 3;20:181. doi: 10.1186/s12909-020-02095-4 (PMC7271445; doi:10.1186/s12909-020-02095-4)
Supplement: Supplementary file 1 — Additional file 1: Supplementary Table 1. 32-item DES questionnaire used in this study. [file 12909_2020_2095_MOESM1_ESM.docx]

**Supplementary Table 1.** 32-item DES questionnaire used in this study

| **Item no.** | **Content** | **Domains of potential stressors** |
| --- | --- | --- |
| 1 | Moving away from home | Social stressors |
| 2 | Lack of home atmosphere |  |
| 3 | Environment in which to study |  |
| 4 | Making friends |  |
| 5 | Intimate Relationships |  |
| 6 | Conflict with spouse/mate over career development |  |
| 7 | Having multiple roles |  |
| 8 | Personal physical health |  |
| 9 | Financial responsibilities |  |
| 10 | Discrimination due to gender or social class |  |
| 11 | Expectation vs reality of dental school | Faculty and administration |
| 12 | Approachability of staff |  |
| 13 | Criticism about academic or preclinical work |  |
| 14 | Rules and regulations of the dental school |  |
| 15 | Amount of cheating in school |  |
| 16 | Lack of input in decision making process in dental school |  |
| 17 | Lack of time for relaxation | Workload |
| 18 | Having reduced holidays compared with other students |  |
| 19 | Amount of assigned course work |  |
| 20 | Lack of time to do assigned school work |  |
| 21 | Learning precision manual skills required for clinical and laboratory work |  |
| 22 | Late ending time/completing graduation requirements |  |
| 23 | Language barrier | Self-efficacy beliefs |
| 24 | Fear of not being able to catch up if falling behind |  |
| 25 | Lack of confidence to be a successful dental student |  |
| 26 | Uncertainty about dental career |  |
| 27 | Lack of confidence to become a successful dentist |  |
| 28 | Difficulty of course work | Performance pressure |
| 29 | Examinations |  |
| 30 | Competition for grades |  |
| 31 | Fear of failing a course of the year |  |
| 32 | Fear of not having possibility to pursue a postgraduate dental education program |  |
